# Non‐Invasive Sampling Reveals Genetic Differentiation and Lineage Structure in the Rare Butterfly *Luehdorfia chinensis* Leech (Lepidoptera Papilionidae)

**DOI:** 10.1002/ece3.73764

**Published:** 2026-06-04

**Authors:** Wen‐Jing Yang, Hui‐dan OuYang, Qi Zhu, Yun‐Hao Zou, Bin Jiang, Shi Xu, Guang‐Ping Huang, Guo‐fang Jiang, Ju‐Ping Zeng

**Affiliations:** ^1^ Jiangxi Provincial Key Laboratory of Conservation Biology, Key Laboratory of National Forestry and Grass and Administration on Forest Ecosystem Protection and Restoration of Poyang Lake Watershed, College of Forestry Jiangxi Agricultural University Nanchang People's Republic of China; ^2^ Lushan Forest Ecosystem Observation Station Lushan National Nature Reserve of Jiangxi Jiujiang People's Republic of China; ^3^ Vocational Normal College Jiangxi Agricultural University Nanchang People's Republic of China; ^4^ Taohongling Sika Deer National Nature Reserve of Jiangxi Pengze People's Republic of China; ^5^ College of Oceanology and Food Science Quanzhou Normal University Quanzhou People's Republic of China; ^6^ Jiulianshan Forest Ecosystem Observation Station Jiulianshan National Nature Reserve of Jiangxi Longnan People's Republic of China

**Keywords:** conservation genetics, geographic isolation, *Luehdorfia chinensis*, non‐invasive sampling (NIS), population structure

## Abstract

Conservation genetics research on rare and legally protected butterflies is often constrained by restrictions on destructive sampling, resulting in longstanding knowledge gaps regarding population structure and evolutionary differentiation. Non‐invasive sampling (NIS) offers a potential solution, yet its reliability for generating conservation‐relevant genetic data in endangered Lepidoptera remains insufficiently evaluated. The feasibility of NIS was assessed in the Chinese endemic butterfly *Luehdorfia chinensis* by comparing DNA yield and quality from naturally shed materials, including larval and pupal exuviae and frass, across developmental stages and preservation methods. All material types yielded amplifiable COI sequences, with fifth‐instar larval exuviae preserved at −20°C or in anhydrous ethanol consistently providing the highest DNA quality, indicating strong suitability for COI‐based population‐level analyses and long‐term monitoring. By integrating NIS‐derived sequences with publicly available COI data from additional populations, two geographically structured genetic lineages were identified, corresponding to low‐altitude populations in the Yangtze River basin (*L. c. chinensis*) and high‐altitude populations in the Qinling Mountains (*L. c. huashanensis*). These lineages were separated by five mutational steps and accounted for 82.9% of among‐group genetic variance (AMOVA), consistent with recognized subspecific boundaries and reflecting the role of mountain–basin topography in limiting gene flow. Lower genetic diversity in populations broadly consistent with *L. c. huashanensis* may indicate potential conservation concern under environmental change and habitat fragmentation. Overall, the results demonstrate that non‐invasive materials can yield robust genetic data for resolving population structure in protected butterflies, supporting their broader application in conservation genetics and management.

## Graphical abstract

Non‐invasive sampling was evaluated as a genetic tool for the rare butterfly *Luehdorfia chinensis* using naturally shed materials. Fifth‐instar larval exuviae provided high‐quality mitochondrial DNA and enabled robust population genetic analyses. Distinct genetic differentiation was detected between Yangtze Basin and Qinling Mountain lineages, reflecting geographic isolation and subspecific structure.

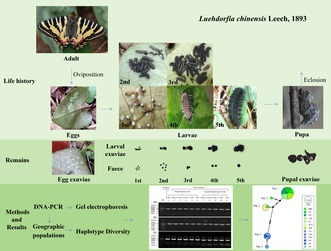

## 1 Introduction

Genetic diversity underpins ecosystem functioning and is central to understanding evolutionary processes at the molecular level (Wang et al. 2017; Schmidt and Garroway 2021; Nayak et al. 2025). However, acquiring genetic material from rare and endangered taxa remains challenging because these species are infrequently encountered and often subject to legal, ethical, or ecological restrictions that preclude invasive sampling. Taberlet and Waits (1998) categorized animal DNA sampling into three types: damaging sampling (DS), non‐damaging sampling (NDS), and non‐invasive sampling (NIS). DS may threaten organismal survival or cause physiological harm, limiting its applicability (Taberlet and Luikart 1999; Rennolds and Bely 2023). In contrast, NDS and NIS minimize impacts on survival and maintain organismal integrity. NDS, however, may still involve invasive instruments, such as biopsy dart guns (Pagano et al. 2014; Mijele et al. 2016), whereas NIS focuses on collecting naturally shed biological materials (e.g., feces, hair, larval and pupal exuviae, sloughed skin cells) during the organism's life history, thereby achieving a truly non‐invasive approach (Schilling et al. 2022).

Rare insects often have small, fragmented populations and cryptic ecologies. Legal protection further constrains invasive sampling, making DNA acquisition via DS or NDS approaches impractical. Naturally produced materials—including egg chorions, larval feces, and larval or pupal exuviae—harbor recoverable genetic material generated throughout insect ontogeny. These materials represent promising sources for non‐invasive DNA recovery, enabling genetic analyses without compromising individual survival or population stability (Pan et al. 2004). For example, high‐quality DNA has been extracted from egg chorions of *Cyclargus thomasi bethunebakeri* W.P. Comstock and Huntington (Feinstein 2004), and ND1 sequences have been amplified from larval exuviae of *Vanessa cardui* (Linnaeus, 1758) (Storer et al. 2019). Similarly, the complete mitochondrial genome of the rare butterfly *Teinopalpus aureus* Mell (Lepidoptera: Papilionidae) has been sequenced from DNA extracted from feces and pupal exuviae using optimized protocols (Zou 2023; Yang et al. 2024). Collectively, these studies demonstrate that NIS is both feasible and efficient, providing reliable genetic data for species‐level analyses (Ferreira et al. 2018; Andrews et al. 2021). Despite these advances, NIS remains underutilized in insect conservation studies, and no standardized framework exists for its systematic application. Expanding NIS‐based DNA extraction to additional rare insect taxa could improve understanding of their genetic diversity and evolutionary dynamics, thereby supporting evidence‐based conservation strategies.

*Luehdorfia chinensis* Leech is a narrowly distributed butterfly species endemic to China (Zhou 1994; Matsumura 2004; Wu and Xu 2017). It is confined to fragmented habitats in the middle and lower Yangtze River Basin and the Qinling Mountains due to specialized ecological requirements (Hu et al. 1992; Yuan et al. 1998; Su 2020; Chen et al. 2022; Chen 2023). It was first listed on the IUCN Red List in 1985 (Jonathan and Craig 2004) and has been designated a nationally protected species (Grade II) under China's List of State Key Protected Wild Animals since 1989. Populations are highly fragmented, geographically isolated, and exposed to demographic and ecological pressures that threaten persistence (Su 2020; Chen et al. 2022; Zou et al. 2023). Consequently, research has increasingly focused on its geographic affinities, intraspecific differentiation, and genetic diversity (Shirozu and Hara 2005; Dong 2015, 2016; Chen et al. 2019). Two subspecies—*L. c. chinensis* and *L. c. huashanensis* (also referred to as *L. chinensis* leei)—were historically distinguished mainly by the shape of the black transverse band on the forewing (Lee and Ito 1982; Hu et al. 1992; Niu et al. 1995; Wu 2001). A third putative subspecies, *L. c. shoui*, has also been proposed (Shou 2013). However, the validity of *L. c. shoui* remains controversial because of weak morphological diagnosability and pronounced ecological niche overlap with *L. c. huashanensis*, including sympatric distribution and shared larval host plants (Wang et al. 2010; Su et al. 2019; Su 2020).

To address these methodological and conservation‐related gaps, we collected various non‐invasive materials from *L. chinensis* , including larval exuviae, feces, and pupal exuviae at different developmental stages. Most samples were obtained from a laboratory‐maintained population at the “Butterfly Conservation and Research Center” in Taohongling Nature Reserve, while additional materials were collected from other geographically distinct populations. Using commercial DNA extraction kits, we evaluated how sample type and preservation method influenced DNA quality, aiming to identify suitable NIS materials for molecular analysis. We further used mitochondrial COI marker to investigate phylogenetic relationships, intraspecific divergence, and the geographic structuring of genetic diversity in *L. chinensis* , an approach widely used in Lepidoptera research (Klečková et al. 2023; Kanthaswamy 2024). The study aimed to identify suitable non‐invasive materials for *L. chinensis* and evaluate their utility for genetic marker analyses and cross‐regional population genetic research.

## 2 Materials and Methods

### 2.1 Non‐Invasive Sampling

**FIGURE 1:**
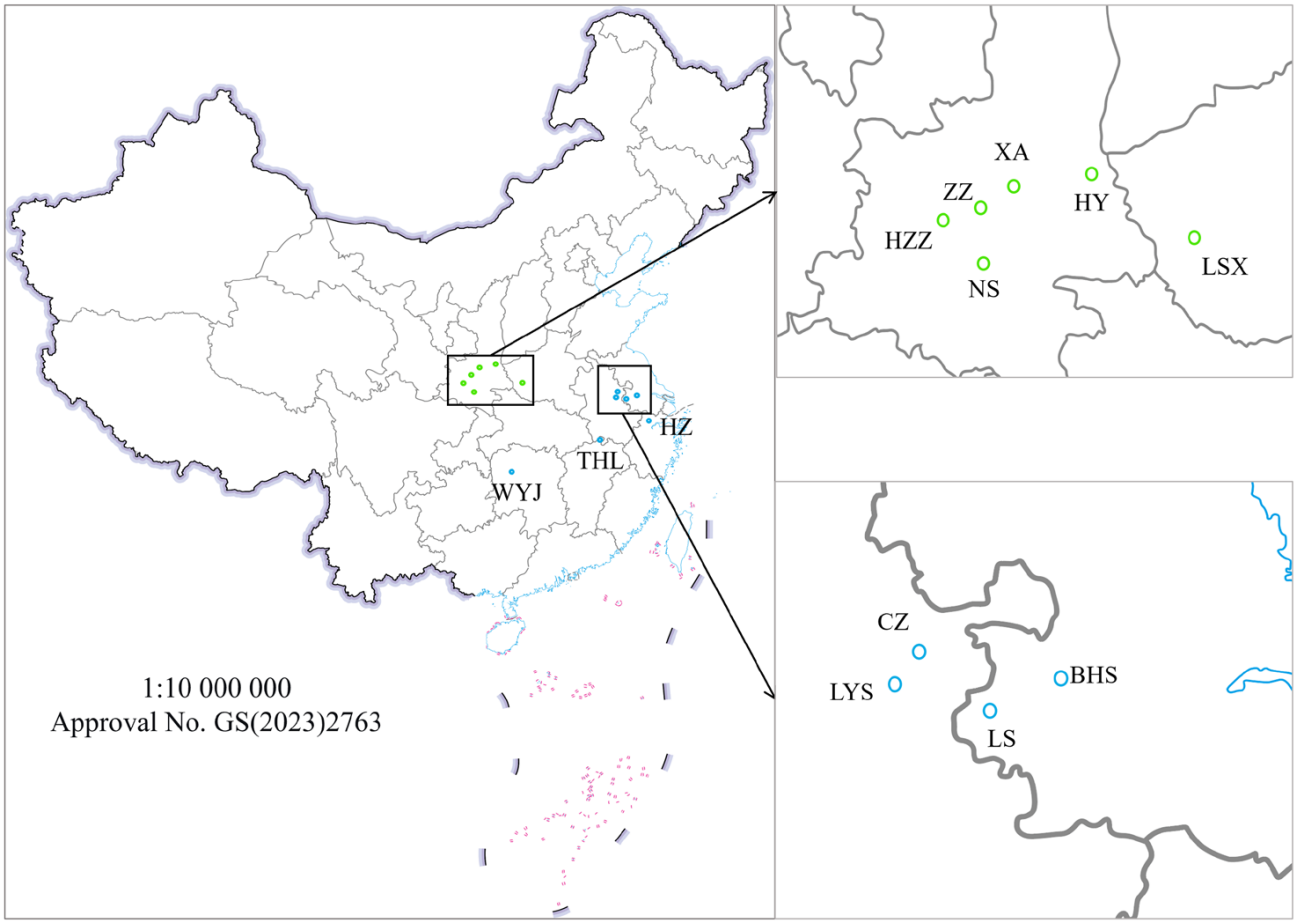
Sampling sites of *L. chinensis* populations. Green dots: Qinling Mountains; blue dots: Middle and lower Yangtze River. Base map obtained under approval number GS (2023)2763.

Larval exuviae of different instars, feces, and pupal exuviae of *L. chinensis* were collected in 2023 from an indoor population maintained at the Butterfly Conservation and Research Center, Taohongling Nature Reserve (THL), according to Yang et al. (2024). Additional larval exuviae were collected from field populations at Laoshan (LS) and Houzhenzi (HZZ) during surveys from 2023 to 2024. Sample numbers for each material type and preservation treatment are summarized in Table 1. For comparative analyses, cytochrome c oxidase subunit I (COI) sequences from populations in Huayin (HY), Zhouzhi (ZZ), Xi'an (XA), Luanshanxian (LSX), Ningshan (NS), Baohuashan (BHS), Chuzhou (CZ), Hangzhou (HZ), Wuyunjie (WYJ), and Langyashan (LYS) were obtained from GenBank. The geographic origins of all sampled and reference populations are shown in Figure 1.

**TABLE 1:** Sources, preservation durations, and methods of non‐invasive sampling materials for *L. chinensis*.

Sampling	Sources	Preservation	Duration^a^	Repetitions
Non‐invasive	3rd larval exuviae (LvE_3)	Ethanol‐preserved	ST	5
			MT	5
			LT	5
		Lyophilization‐preserved	ST	5
			MT	5
			LT	5
	4th larval exuviae (LvE_4)	Ethanol‐preserved	ST	5
			MT	5
			LT	5
		Lyophilization‐preserved	ST	5
			MT	5
			LT	5
	5th larval exuviae (LvE_5)	Ethanol‐preserved	ST	5
			MT	5
			LT	5
		Lyophilization‐preserved	ST	5
			MT	5
			LT	5
	Larval feces (LvF)	Ethanol‐preserved	ST	5
			MT	5
			LT	5
		Lyophilization‐preserved	ST	5
			MT	5
			LT	5
	Pupal exuviae (LvP)	Ethanol‐preserved	ST	5
			MT	5
			LT	5
		Lyophilization‐preserved	ST	5
			MT	5
			LT	5
Damaging	Larval head (LvH)	Ethanol‐preserved	ST	5

^a^ LT, long‐term preserved (> 12 months); MT, mid‐term preserved (5–7 months); ST, Short‐term preserved (< 1 month). “Repetitions” refers to the number of independent samples rather than repeated analyses of the same sample.

Sampling materials were stored in 2 mL centrifuge tubes and preserved either in anhydrous ethanol or by lyophilization, with short‐term (< 1 month), medium‐term (5–7 months), or long‐term (> 12 months) storage (Table 1). Larvae that died prematurely during indoor rearing at THL were preserved in absolute ethanol and used as controls in DNA extraction experiments.

### 2.2 DNA Extraction and Detection

DNA from fecal samples was extracted using the TIANamp Stool DNA Kit, with approximately 0.18–0.2 g of feces processed following the manufacturer's instructions. Fecal samples were collected from individually reared larvae and processed as independent units. DNA from other NIS materials (larval exuviae and pupal exuviae) was extracted using the HiPure Insect DNA Kit. All larval instars and pupal exuviae were dissected and standardized to ~10 mg prior to extraction. Each sample was placed in a 1.5 mL centrifuge tube with 250 μL Buffer ITL and 20 μL proteinase K and incubated at 55°C for 6 h. For fecal samples (180–220 mg), DNA was finally eluted in 50 μL of TB buffer according to the TIANamp Stool DNA Kit protocol, whereas DNA from other NIS materials was eluted in 20 μL of Buffer TE using the HiPure Insect DNA Kit. Total DNA yield was calculated as DNA concentration (ng/μL) × elution volume (μL). DNA concentration and purity were quantified using a Nanodrop spectrophotometer and subsequently used for PCR amplification of the mitochondrial COI gene.

### 2.3 PCR Amplification

PCR amplification of the mitochondrial COI gene was performed in a 50 μL reaction using primers 5′–3′: forward, ATTCAACCAATCATAAAGATAT; reverse, TAAACTTCTGGATGTCCAAAAA, yielding a 623 bp fragment. The PCR cycling protocol consisted of an initial denaturation at 95°C for 5 min, followed by 35 cycles of 95°C for 30 s, 51°C for 30 s, and 72°C for 30 s, with a final extension at 72°C for 10 min. Reactions were held at 4°C until further processing. PCR products were verified on a 1% TAE agarose gel containing Super GelRed using a DYY‐2C electrophoresis apparatus (Beijing LiuYi Biotechnology Co. Ltd.). Gel images were captured with a CLiNX imaging system (Shanghai Qinxing Scientific Instrument Co. Ltd.), and successfully amplified products were sequenced bidirectionally by Sanger sequencing at Shanghai Bioengineering Co. Ltd.

### 2.4 Sequencing Data Processing and Gene Alignment Submission

Sequencing quality was assessed using DNASTAR SeqMan (Burland 2000). The obtained sequences were confirmed as COI sequences by BLAST searches against the NCBI database and then aligned with reference sequences from GenBank in MEGA 7 (Kumar et al. 2016). Additional COI sequences from geographic populations (CZ1–5, BHS1–6, HZ1–5, HY, ZZ, LYS, LSX, WYJ, THL1) were obtained from GenBank for comparative analyses.

### 2.5 Mitochondrial Haplotype Diversity and Demographic History

Mitochondrial COI sequences from non‐invasive samples and public databases were aligned using ClustalW in MEGA 7 (Kumar et al. 2016). Haplotype identification and calculations of genetic diversity indices, including haplotype diversity (Hd) and nucleotide diversity (π), were performed in DnaSP v6.0 (Rozas et al. 2017). A haplotype network was generated using the median‐joining method in PopART v1.7 (Leigh et al. 2015). Population differentiation was further assessed using AMOVA in Arlequin v3.5 (Excoffier and Lischer 2010). A Bayesian phylogenetic tree was reconstructed using PhyloSuite under the GTR + F + I substitution model, which was selected as the best‐fit model according to ModelFinder implemented in PhyloSuite (Xiang et al. 2023).

### 2.6 Data Analysis

Variations in genomic DNA concentration and purity across preservation methods, storage durations, and sample sources were assessed using multifactorial ANOVA. Tukey's test was used for post hoc comparisons. Statistical analyses were performed using SPSS 22.0, with significance set at *p* < 0.05. Data were visualized using OriginPro 2023b (Stevenson 2011).

## 3 Results

### 3.1 Effects of Noninvasive Sampling Materials and Storage Conditions on DNA Quality

The quality of genomic DNA extracted from non‐invasive samples (NIS) was significantly influenced by sample type, preservation method, and storage duration. Multivariate analyses showed that DNA concentration was strongly affected by material source (*p* < 0.0001), preservation duration (*p* < 0.0001), preservation method (*p* < 0.0001), and the interactions between source × preservation method (*p* = 0.016) and source × preservation duration (*p* = 0.032) (Table 2). In contrast, DNA purity was primarily affected by sample type (*p* < 0.0001), with minimal effects of preservation method (Table 3). These results indicate that the choice of NIS material is the most critical factor for obtaining high‐quality DNA, while storage conditions play a secondary but significant role.

**TABLE 2:** Effects of sample source, preservation method, and duration on genomic DNA concentration (multivariate analysis).^a^

Factor	Type II sum of squares	df	Mean square	*F*	*p*
Corrected model	631,693.475^b^	32	19,740.421	110.637	< 0.0001
Intercept	514,487.424	1	514,487.424	2883.501	< 0.0001
Sources	537,085.173	5	107,417.035	602.031	< 0.0001
Preservation	1241.536	1	1241.536	6.958	0.009
Duration	25,250.522	2	12,625.261	70.760	< 0.0001
Sources × preservation	2256.633	4	564.158	3.162	0.0160
Sources × duration	3670.442	10	367.044	2.057	0.0320
Preservation × duration	163.471	2	81.735	0.458	0.6330
Error	23,552.041	132	178.425		
Total	1,169,732.940	165			
Corrected total	655,245.516	164			

^a^ With sources and preservation as fixed variables and preserved duration as a covariate.

^b^ *R* ^2^ = 0.964 and adjust *R* ^2^ = 0.955.

**TABLE 3:** Effects of sample source, preservation method, and duration on genomic DNA quality (multivariate analysis).^a^

Factor	Type II sum of squares	df	Mean square	*F*	*p*
Corrected model	1.799^b^	32	0.056	4.939	< 0.0001
Intercept	493.690	1	493.690	43,377.687	< 0.0001
Sources	1.252	5	0.250	22.005	< 0.0001
Preservation	0.019	1	0.019	1.637	0.203
Duration	0.019	2	0.010	0.844	0.432
Sources × preservation	0.088	4	0.022	1.928	0.110
Sources × duration	0.124	10	0.012	1.092	0.373
Preservation × duration	0.002	2	0.001	0.095	0.910
Error	1.502	132	0.011		
Total	496.991	165			
Corrected total	3.301	164			

^a^ With sources and preservation as fixed variables and preserved duration as a covariate.

^b^ *R* ^2^ = 0.545 and adjust *R* ^2^ = 0.435.

**FIGURE 2:**
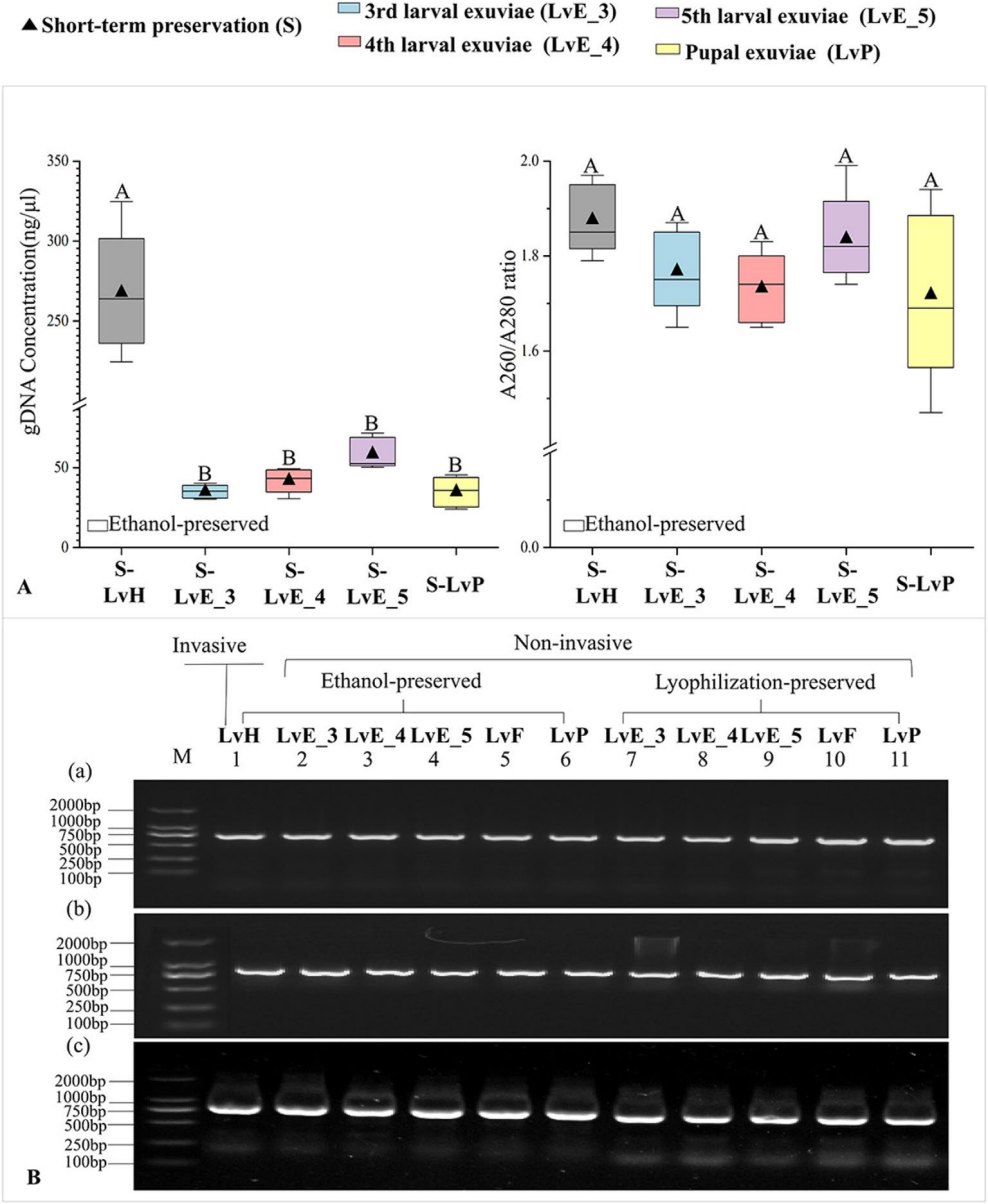
DNA quality and PCR amplification performance of destructive and non‐invasive samples under different preservation conditions. (A) DNA quality comparison between DS (LvH: larval head) and NIS (LvE_3–5: 3rd–5th instar larval exuviae; LvP: pupal exuviae) under identical treatments. Uppercase letters indicate significant differences among preservation methods within the same duration. (B) Gel electrophoresis of COI PCR products from NIS samples. M: DNA marker; (a) short‐term (< 1 month), (b) mid‐term (5–7 months), (c) long‐term (> 12 months).

**FIGURE 3:**
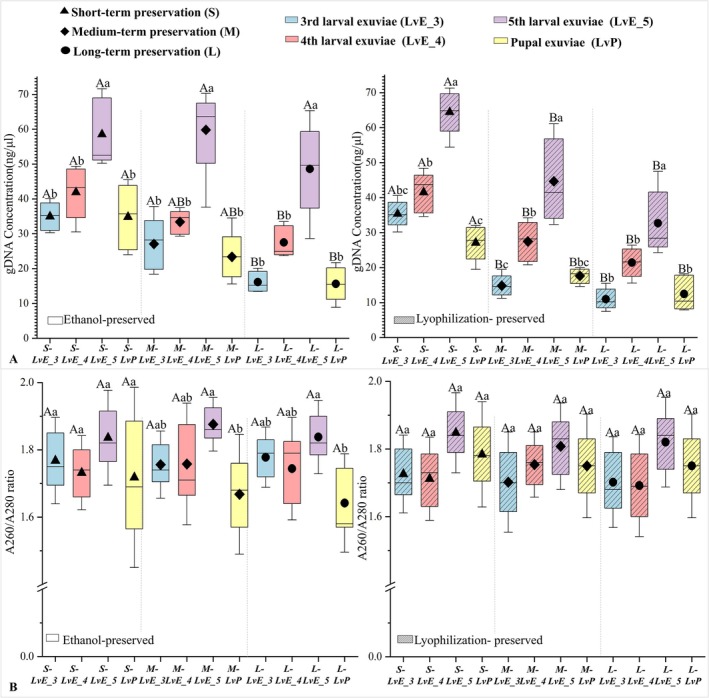
Comparison of genomic DNA concentration and purity in various NIS samples under different preservation treatments. (A) Genomic DNA concentration of NIS samples across treatments and storage durations. (B) Genomic DNA purity (A260/280) of NIS samples under different preservation treatments. Uppercase letters indicate significant differences between preservation methods within the same storage duration, whereas lowercase letters indicate significant differences across storage durations within the same preservation method.

All NIS materials yielded amplifiable DNA. Although DNA concentrations were lower than that of the destructive control (267.8 ± 37.6 ng/μL), the highest concentration among NIS materials was observed in fifth‐instar larval exuviae (58.54 ± 9.72 ng/μL, Figure 2A), DNA purity did not differ significantly among sample types (one‐way ANOVA: *F*_4_,_20_ = 1.965, *p* = 0.139). Electrophoresis confirmed successful PCR amplification across all NIS materials, including feces (Figure 2B). Among the tested sources, fifth‐instar larval exuviae (LvE_5) produced the highest DNA concentration (58.54 ± 9.72 ng/μL) and the most consistent purity values (Figure 3), suggesting they are the optimal material for non‐invasive DNA recovery. Total DNA yield followed the same trend as DNA concentration, with destructive samples yielding the highest amounts (4334.4–5356.8 ng). Among non‐invasive materials, fifth‐instar larval exuviae performed well (653.6–1288.8 ng), fecal samples were more variable (Data S2).

**FIGURE 4:**
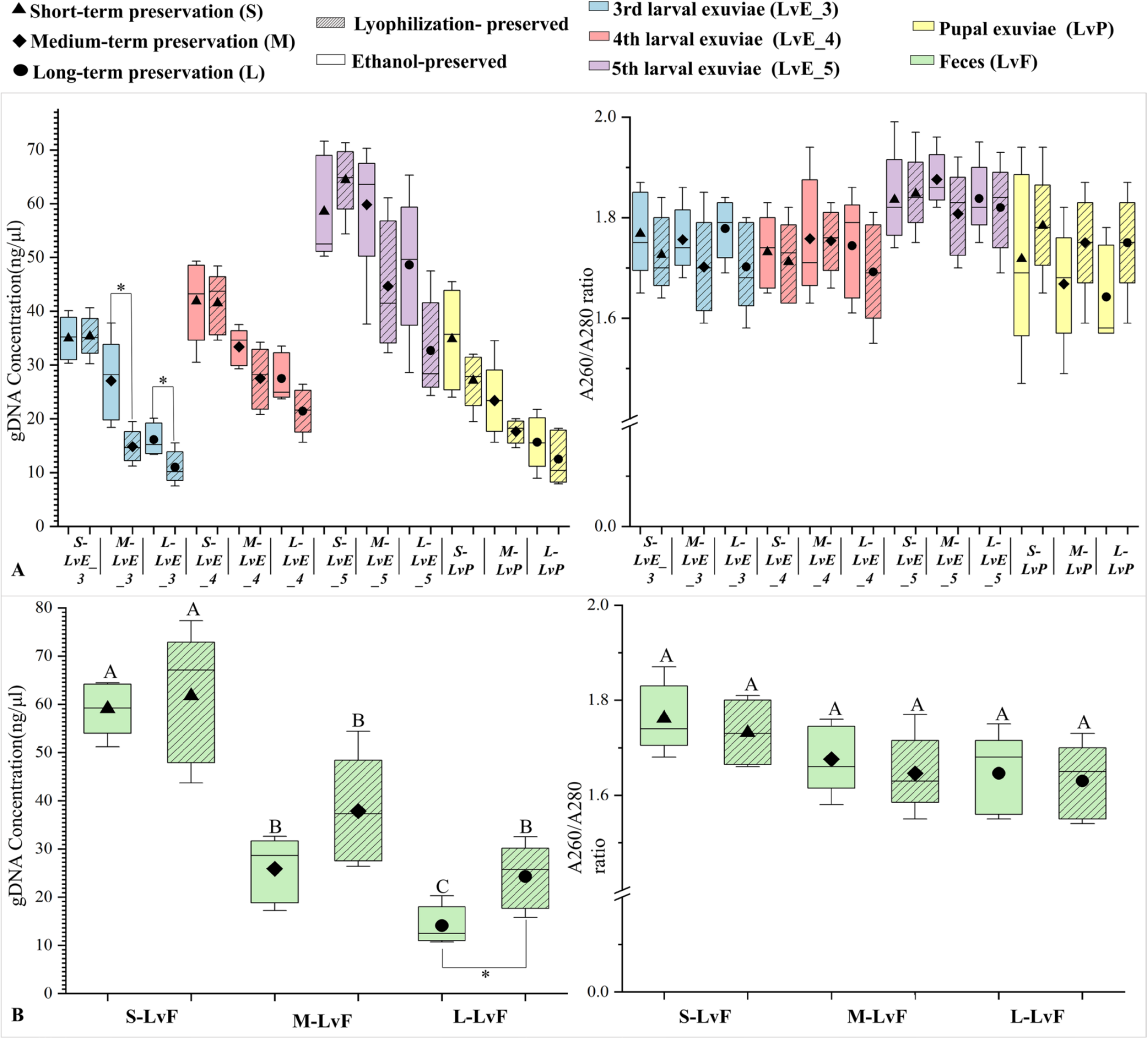
DNA quality of non‐invasive sampling (NIS) materials across preservation methods and durations. (A) DNA concentration (left) and purity (right) of different NIS sample types under each preservation method. Asterisks indicate statistically significant differences (*p* < 0.05). (B) DNA concentration (left) and purity (right) of fecal (LvF) samples across preservation durations and methods. Uppercase letters indicate significant differences among sample types within the same preservation duration, whereas lowercase letters indicate significant differences among preservation durations within the same sample type.

Storage duration negatively affected DNA quality. Samples stored for over 12 months exhibited lower DNA concentrations compared to short‐term preserved samples (Figure 3A). Ethanol‐preserved samples showed declines in DNA purity under medium‐ and long‐term storage (*F*_3,16_ = 4.070 and 4.171, *p* < 0.05; Figure 3B). Electropherograms revealed hetero‐bands and smearing in medium‐ and long‐term preserved samples, whereas short‐term samples produced clean and distinct bands (Figure 2B). Preservation efficacy varied by material type: for non‐fecal samples, ethanol preservation yielded higher DNA concentrations than lyophilization under medium‐ and long‐term storage (Figure 4A), whereas for lyophilization was superior to ethanol after long‐term storage (*t* = −2.843, *p* < 0.05; Figure 4B). DNA purity, however, did not differ significantly between preservation methods.

### 3.2 Genetic Diversity and Population Differentiation of *L. chinensis*

**FIGURE 5:**
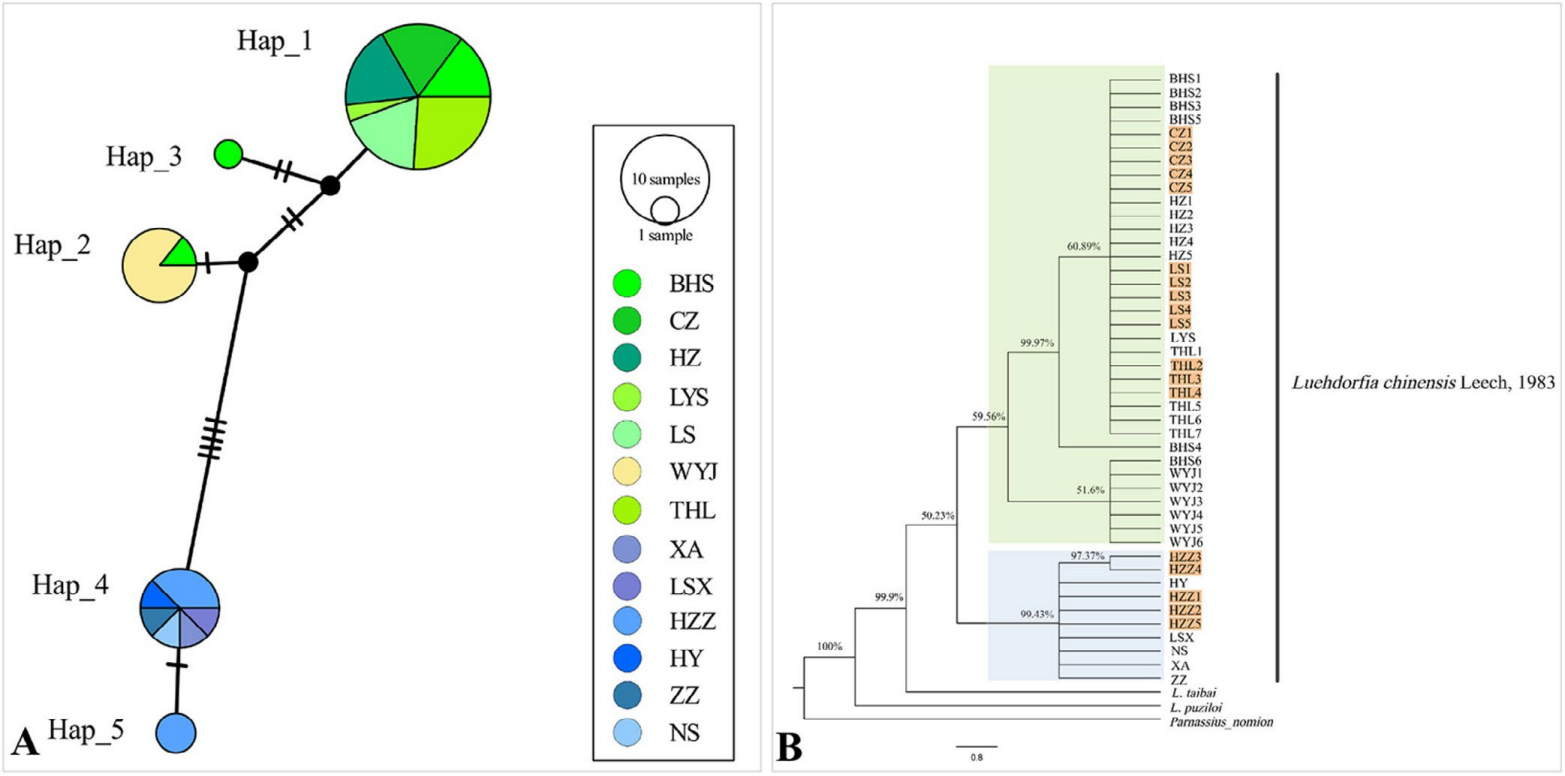
COI‐based phylogeographic structure of *L. chinensis* across its range. (A) Median‐joining haplotype network showing five haplotypes grouped into two major haplogroups: haplogroup A (Hap_1—Hap_3), mainly distributed in the Yangtze River basin, and haplogroup B (Hap_4—Hap_5), mainly distributed in the Qinling Mountains. Black nodes indicate inferred intermediate haplotypes, and perpendicular hatch marks on the connecting lines indicate mutational steps. (B) Bayesian inference tree based on COI sequences under the GTR + F + I model, showing the same bipartite pattern recovered in the haplotype network. Sequences generated from non‐invasive sampling (NIS) are highlighted in orange. Node support values indicate posterior probabilities.

Analysis of COI sequences from 13 geographic populations resolved five haplotypes (Hap_1–Hap_5), which formed two geographically structured haplogroups (Figure 5A): Haplogroup A comprised Hap_1–Hap_3, and haplogroup B, comprising Hap_4–Hap_5. Haplogroup A was mainly distributed in BHS, CZ, HZ, LS, THL, WYJ, and LYS, whereas haplogroup B was mainly distributed in HY, ZZ, XA, LSX, NS, and HZZ. The five mutational steps separating the two haplogroups suggest a clear genetic discontinuity rather than gradual differentiation across the range. Bayesian phylogenetic reconstruction recovered the same bipartite pattern, with both lineages forming distinct clades (Posterior support = 0.59; Figure 5B), and AMOVA attributed 82.9% of the total molecular variance to divergence between these two predefined geographic groups: Group I (BHS, CZ, HZ, LS, THL, WYJ, LYS) and Group II (HY, ZZ, XA, LSX, NS, HZZ) (Table 4).

**TABLE 4:** AMOVA of distinct populations of *L. chinensis* based on multiple genes.

Source of variation	Sum of squares	Variance components	Percentage variation
Among groups	54.108	3.35398	82.92059
Among populations within groups	19.814	0.48145	11.90301
Within populations	6.7	0.20937	5.17639
Total	80.622	4.04481	

Genetic diversity metrics further supported this pattern of population structuring. Group I exhibited higher haplotype and nucleotide diversity (Hd = 0.3748; π = 0.00236) than Group II (Hd = 0.3556; π = 0.00057) (Table 5). However, neutrality tests were not significant in either group and therefore provide only limited support for demographic interpretation in the present dataset.

**TABLE 5:** Genetic diversity and neutrality test results of population groups based on COI sequences.

Group	Genetic diversity		Neutral test	
	Hd	π	Tajima's *D*	Fu's *F*s
Group I	0.3748	0.00236	0.01624	2.958 (*p* > 0.1)
Group II	0.3556	0.00057	0.01499	0.417 (*p* > 0.1)

## 4 Discussion

Conservation genetics of rare and range‐restricted butterflies is frequently constrained by limited opportunities for specimen acquisition. Endangered taxa often occur in isolated habitats and at low population densities, where conventional destructive sampling is either impractical or prohibited by legal protections (Brockerhoff et al. 2023; Dwivedi et al. 2025). Under such circumstances, genetic information essential for conservation planning remains scarce, ultimately hindering the assessment of population viability, connectivity, and adaptive potential (Schilling et al. 2022; Graham 2022; Ascenzi et al. 2025). Although NIS has been proposed as an alternative in Lepidoptera (Kavanaugh et al. 2014; Tan and Dominy 2018; Yang et al. 2024), the reliability of DNA obtained from different non‐invasive materials and the preservation protocols needed to ensure analytical quality have not been systematically validated. This methodological gap has reduced the feasibility of applying NIS‐derived genetic data to conservation decision‐making for threatened butterfly species.

This study addresses this limitation by systematically evaluating multiple categories of non‐invasive materials from *L. chinensis* . By comparing DNA yield and integrity across developmental sources and preservation treatments, we identified fifth‐instar larval exuviae as the most reliable material for downstream applications. Ethanol preservation generally performed better for larval and pupal exuviae, whereas lyophilization was more effective for fecal samples under long‐term storage. This pattern is consistent with biological expectations: larger body size increases residual cellular material on the inner cuticle (Nguyen et al. 2017; Campli et al. 2024), while ethanol suppresses enzymatic degradation and microbial proliferation (Anchordoquy and Molina 2007; Zimmermann et al. 2008). Lyophilization was particularly effective for fecal samples, likely due to the strong dehydration efficiency that reduces microbial nuclease activity (Hammer et al. 2015; Zhou et al. 2018). These results confirm that NIS can generate DNA of sufficient quality for population‐level analyses if material type and preservation method are appropriately matched (Scriven et al. 2013; Inoda et al. 2015; Baus et al. 2019; Nagarajan et al. 2020; Yang et al. 2024).

Based on the tested NIS materials, we successfully recovered COI sequences from field populations and integrated them with publicly available datasets to resolve the geographic structure of *L. chinensis* . All analyses converged on a consistent pattern of geographic differentiation between low‐altitude populations in the Yangtze basin (Group I: BHS, CZ, HZ, LS, THL, WYJ, LYS) and high‐altitude populations in the Qinling Mountains (Group II: HY, ZZ, XA, LSX, NS, HZZ). The separation of the two haplogroups by five mutational steps, together with AMOVA partitioning (82.9% of variation between groups), and phylogenetic reconstruction, supports clear geographic structuring between the two groups. The absence of shared haplotypes between the two groups is also consistent with restricted connectivity. This pattern is broadly consistent with the historically recognized subspecies *L. c. chinensis* and *L. c. huashanensis* and with geographic separation between the Qinling Mountains and the Yangtze River basin (Lee and Ito 1982; Hu et al. 1992; Wu 2001). The putative third subspecies, *L. c. shoui*, remains hypothetical and is not recognized in the current fauna treatment. Our results are instead broadly consistent with, and provide additional support for, the traditional subdivision of *L. chinensis* into *L. c. chinensis* and *L. c. huashanensis*. Together, these results suggest that NIS‐derived mitochondrial data can help identify lineage boundaries associated with regional geographic structuring.

High‐altitude populations are often exposed to strong selective pressures, which can drive rapid adaptive responses in complex environments (Halbritter et al. 2015; Klečková et al. 2023; Wang et al. 2024). Consequently, these populations have become a focal point in mountain ecosystem ecology and evolutionary research (Lozier et al. 2021; Zhao et al. 2023). However, increasing elevation also intensifies geographic isolation, restricting gene flow and promoting population differentiation (Hodkinson 2005; Kleivane et al. 2022). The genetic differentiation of *L. c. huashanensis* may be related to geographic separation associated with the Qinling Mountains. Prolonged isolation may reduce genetic diversity in high‐altitude populations, potentially leading to the loss of adaptive alleles (Schiffers et al. 2013; McCulloch et al. 2022).

Beyond physical isolation, ecological factors such as host plant specialization may also be relevant to population differentiation, particularly in specialist species. In the Qinling Mountains, larvae of *L. chinensis* primarily feed on *Asarum sieboldii*, whereas populations along the middle and lower Yangtze River basin mainly utilize *A. forbesii* (Yuan et al. 1998; Wang et al. 2010; Su et al. 2019). Because *A. sieboldii* occurs predominantly at higher altitudes and *A. forbesii* at lower elevations, host plant specialization may also be associated with reinforcing genetic divergence (Zhang et al. 2015; Jones et al. 2022).

Together, these findings highlight the subtle genetic differentiation and potential vulnerability of high‐altitude populations to environmental changes and extreme climatic events (Pironon et al. 2016; John et al. 2024). Conservation strategies should therefore take enhancing gene flow and mitigating genetic bottlenecks through measures such as habitat preservation, ecological corridor construction, and population monitoring, while broader genomic and ecological data will be needed to evaluate connectivity and conservation vulnerability more fully (Dalui et al. 2024; Gagnaire 2020).

## 5 Conclusion

NIS represents a reliable and conservation‐compatible approach for population genetic analyses of rare butterflies. COI sequences obtained from naturally shed materials revealed two geographically structured groups of *L. chinensis* , broadly consistent with *L. c. chinensis* in the Yangtze River basin and *L. c. huashanensis* in the Qinling Mountains, and consistent with geographic structuring associated with topographic separation. The lower genetic diversity observed in high‐altitude populations may indicate potential conservation concern under environmental change, emphasizing the need for targeted conservation management. Overall, non‐invasive genetic approaches provide an effective framework for COI‐based population structure inference and conservation planning in protected and range‐restricted insect species, while their broader genomic applications require further validation.

## Author Contributions

**Wen‐Jing Yang:** data curation (equal), investigation (equal), methodology (equal), resources (equal), software (equal), visualization (equal), writing – original draft (equal), writing – review and editing (equal). **Hui‐dan OuYang:** formal analysis (equal), software (equal), writing – review and editing (equal). **Qi Zhu:** investigation (equal). **Yun‐Hao Zou:** formal analysis (equal). **Bin Jiang:** conceptualization (equal). **Shi Xu:** investigation (equal), resources (equal). **Guang‐Ping Huang:** conceptualization (equal), writing – review and editing (equal). **Guo‐fang Jiang:** writing – review and editing (equal). **Ju‐Ping Zeng:** conceptualization (equal), formal analysis (equal), funding acquisition (equal), investigation (equal), project administration (equal), resources (equal), validation (equal), visualization (equal), writing – original draft (equal), writing – review and editing (equal).

## Funding

This work was supported by the National Natural Science Foundation of China (Grant no. 32360272), the Science and Technology Innovation Project of Jiangxi Forestry Bureau (no. 202233), and the Jiangxi Provincial Key Laboratory of Conservation Biology (no. 2023SSY02081).

## Ethics Statement

Ethical approval was not required as no live animals were harmed during the collection of non‐invasive samples. All sampling was conducted under the approved project of the Taohongling Sika Deer National Nature Reserve of Jiangxi (Project No. 2024JXAUHX049), ensuring compliance with national regulations for the protection of Grade II protected species.

## Consent

The authors have nothing to report.

## Conflicts of Interest

The authors declare no conflicts of interest.

## Supporting information


**Data S1:** ece373764‐sup‐0001‐DataS1.zip.

## Data Availability

The data supporting the findings of this study are provided as Supporting Information files (Data S1–S6), including COI sequence files, population genetic analysis input files, haplotype data, DNA quality measurements, and sample metadata. Newly generated COI sequences have been deposited in GenBank, with accession numbers provided in Table 6. GenBank accession numbers of COI sequences from geographic populations of *L. chinensis*. From the NCBI database.
